# Preface to parasites of the genital tract: short- and long-term consequences

**DOI:** 10.1017/S0031182025101303

**Published:** 2025-12

**Authors:** J. Russell Stothard, Sekeleghe A. Kayuni, Janelisa Musaya, John T. Ellis

**Affiliations:** 1 Department of Tropical Disease Biology, Liverpool School of Tropical Medicine, Pembroke Place, Liverpool, L3 5QA, UK; 2 Malawi Liverpool Wellcome Programme, Kamuzu University of Health Sciences, Queen Elizabeth Central Hospital Campus, Blantyre 3, Malawi; 3 Pathology Department, School of Medicine and Oral Health, Mahatma Gandhi Campus, Blantyre 3, Malawi; 4 Faculty of Science, School of Life Sciences, University of Technology Sydney, Sydney, NSW, Australia

**Keywords:** gender, HIV, HPV, schistosomiasis, sex, sexual and reproductive health, trichomoniasis

## Abstract

Dioecious species that reproduce by internal fertilization typically carry an associated risk of exposure to sexually transmitted parasites and pathogens. When hosts intermingle for procreation, certain protist and helminth parasites, for example, transfer successfully between individuals and then navigate across various life history traits of their hosts, often probing dimensions in both sex and gender, respectively. In humans, there are many sexually transmitted infections as well as sexually transmitted diseases. A well-known sexually transmitted infection is the flagellated protist *Trichomonas vaginalis* that causes trichomoniasis, with over 150 million new cases reported annually. By contrast, the schistosome blood fluke *Schistosoma haematobium*, though not a sexually transmitted infection, causes significant damage to the male and female genital tracts. Such overt damage raises risks of spreading and acquiring Human Immunodeficiency Virus and Human Papilloma Virus. In Africa, over 50 million women continue to suffer from female genital schistosomiasis, alongside a poorly quantified global burden of travel-related infections. In conjunction with male genital schistosomiasis, urogenital schistosomiasis causes much suffering, within and between afflicted households, inclusive of stigmatization. Both trichomoniasis and schistosomiasis expose several public health needs currently addressed inadequately by routine sexual and reproductive health services. This preface to the *Parasitology* Special Issue entitled ‘*Parasites of the genital tract: short- and long-term consequences*’, introduces 19 papers that explore the short – and long-term impacts of parasitic infections within the genital tract. While current parasitological research is weighted towards human medicine, we encourage future studies that explore veterinary contexts and analogous parasitic diseases within wildlife.

## Graphical abstract

**Figure:**
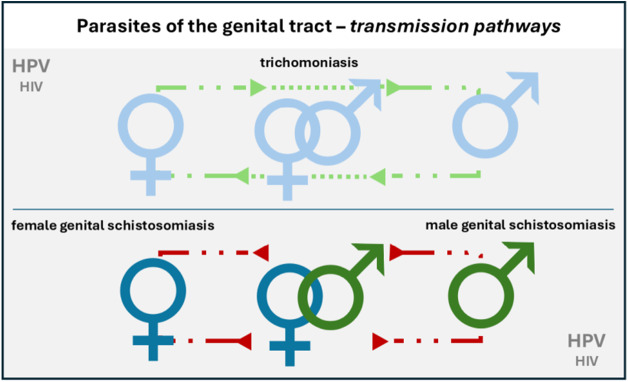

## Introduction

The impetus for this Special Issue of *Parasitology* arose from the authors’ academic curiosities, field experiences and research interests to better understand the transmission and impact of parasites that have evolved to spend all, or part, of their lives within the genital tract of animals. These specialist parasites don’t operate in isolation, for they are sometimes found in association with other more notorious human pathogens. In addition, such parasites and pathogens can have detrimental effects on the *in utero* development of the foetus or induce ill health of the newborn, as (in)directly mediated by poor maternal health and child welfare. Over time, these infection dynamics and disease burdens can be crucial coevolutionary drivers, balancing host life-history traits against the virulence of parasites (Ashby, 2020).

From a global health perspective, there is a long and extensive history of research and control of bacterial and viral pathogens that cause venereal disease in people (Bosire and Akech, 2024), more correctly known today as sexually transmitted infections (STIs) and sexually transmitted diseases (STDs). Across the world, four major STIs – chlamydia, gonorrhoea, syphilis and trichomoniasis – which are each treatable with various prescribed or over-the-counter medicines, detrimentally impact the lives of some 374 million people annually (WHO, 2025). Of note, both sex (biological) and gender (socio-cultural) are important factors in infection risk and disease outcomes; for example, some people can be asymptomatic while others are not, with their well-being curtailed (Bosire and Akech, 2024).

## Parasites that live within the genital tract

Trichomoniasis is caused by the flagellated protist *Trichomonas* (*T.*) *vaginalis*, which is spread through coitus directly or through other sexual contacts, inclusive of vaginal, oral and/or anal intercourse; this parasite is regarded as the most common, curable, non-viral sexually transmitted infection across the world, with some 150 million new cases annually (Muzny et al., 2025). These numbers are likely underestimates, for more broadly, the spectres of criminalization, stigma and shame loom, individually or collectively, over many STIs, typically hampering accurate surveillance, case reporting and effective control (Bosire and Akech, 2024).

In the territory of the World Health Organization-African Regional Office (WHO-AFRO), there is another disease, schistosomiasis, which is much underappreciated in its importance within sexual health and reproductive wellbeing. For example, the WHO-AFRO consultation ‘*Framework for an integrated multisectoral response to TB, HIV, STIs and Hepatitis in the WHO African Region 2021–2030*’, did not mention female genital schistosomiasis (FGS) or male genital schistosomiasis (MGS), despite prior high-level policy evidence about the former as presented in 2019 within the United Nations Programme on HIV/AIDS (UNAIDS) and WHO joint report entitled ‘*No more neglect – FGS and HIV – Integrating sexual and reproductive health interventions to improve women’s lives*’ (UNAIDS, 2019).

**Figure 1.**
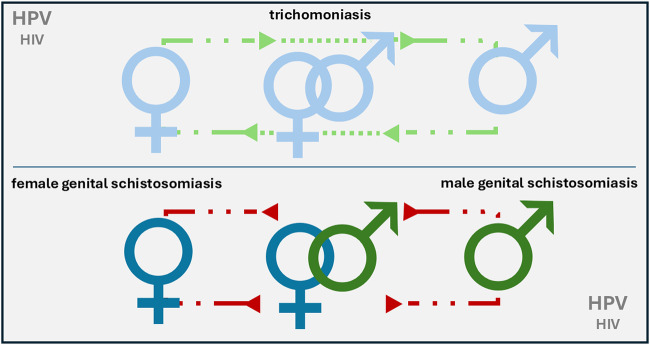
A schematic to illustrate and compare the transmission pathways of trichomoniasis and schistosomiasis. Trichomoniasis is a communicable disease that can transfer from person to person upon coitus or close sexual contact whereas schistosomiasis cannot. Note that it is possible for an individual to have trichomoniasis, schistosomiasis, HIV and HPV, concurrently.

The simple and underlying reason for this is that the parasite responsible, *Schistosoma haematobium*, a trematode blood fluke, is not transmitted person-to-person via sexual routes, hence it falls outside the routine agenda and teaching curriculum of sexual and reproductive health (SRH) services, unless addressed within tropical medicine, where neglected tropical diseases (NTDs) are considered specifically. The schematic of Figure 1 attempts to compare the transmission routes for trichomoniasis and urogenital schistosomiasis (UGS) between the sexes, noting that person-to-person transmission of schistosomiasis is not possible. Of particular note here, each parasitic disease can interact, in sex-biased and gender-biased ways, with other pathogens, notably Human Immunodeficiency Virus (HIV) and Human Papilloma Virus (HPV). Association with high-risk HPV genotypes is now well known (Mukerebe et al., 2025), with an indication of cervical dysplasia too (Rafferty et al., 2021), which is all the more important since *S. haematobium* is a Group I carcinogen (definitely cancerogenic to humans), a well-known instigator of squamous cell bladder cancer (Buonfrate et al., 2025).

## Schistosomes damage the genital tract

**Figure 2.**
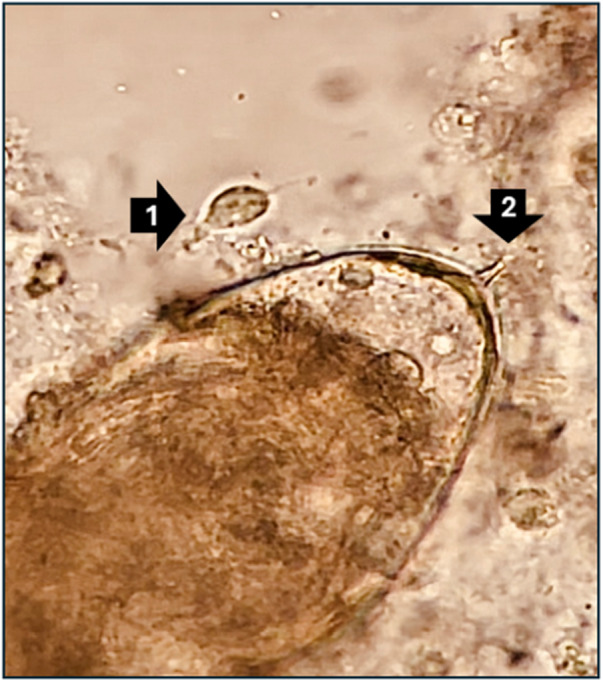
A light micrograph at ×400 magnification of a trophozoite of *Trichomonas vaginalis* (*arrow 1*) adjacent to an egg of *S. Haematobium* with its terminal egg-spine (*arrow 2*) within a cervicovaginal lavage sample from a Malawian woman. There is a short video within supplemental files showing the movement of the trophozoite’s flagella and vitelline fluid surrounding the schistosome miracidium within its eggshell as it starts to hatch.

Typical of all schistosomes, the ephemeral waterborne larvae of these worms, as released from infected aquatic snails, gain entry into the body via percutaneous routes, sometimes also through the buccal mucosa (Buonfrate et al., 2025). Once inside the body, mature adult female worms release copious numbers of cytotoxic and immunogenic eggs into the bloodstream. Some of these exit the body in the urine, via egg-mediated perforations through the bladder wall. The cardinal sign of UGS, *viz.* infection with *S. haematobium*, is macro-haematuria, visual blood in the urine, leaked from the egg-mediated perforations and ancillary immunopathological damage. Coincidentally, this sign overlaps with several STDs and is often erroneously understood by its sufferers, either male or female, and even health professionals, as being a malevolent STI (Kukula et al., 2019; Bustinduy et al., 2022). At the same time, equivalent egg-mediated perforations and later host granulomatous responses occur within the genital tract, for example, abraded and/or fibrotic cervicovaginal surfaces or visible blood within ejaculates. Such sequelae create features that continue to confound routine STI diagnostics and challenge disease surveillance in primary care. Of note here, simple light microscopy can visualize eggs of *S. haematobium* and trophozoites of *T. vaginalis*, as shown in Figure 2, detection of such coinfections is not routinely offered even if diagnostic microscopy is available within the local health facility.

In October 2009, the terminology of UGS became preferred over its original designation of urinary schistosomiasis. This was to highlight its specific damage to the host’s genital tract, giving rise to MGS and FGS terminologies and classifications, each having bearing on increased acquisition and raised transmission of HIV (Mbabazi et al., 2011; Kayuni et al., 2023). In terms of women’s health, FGS is implicated but largely overlooked within a wider variety of SRH issues, from subfertility and infertility to secure menstrual hygiene management, with the latter specifically recognizing a woman’s vulnerability if her safe water, sanitation and hygiene (WASH) needs remain unfulfilled (Kjetland et al., 2012; Stothard, 2012; Christinet et al., 2016; Bustinduy et al., 2022). Put simply, poor maternal health has several wider detrimental effects, from inadequate child care and support through to personal impoverishment for employment and reduced economic capacity within the family household.

Another unusual feature of UGS, which has grown in importance recently, is the zoonotic transmission potential of *S. haematobium*, and its associated hybrids or introgressed variants with *Schistosoma bovis* or *Schistosoma mattheei* (Léger et al., 2020; Juhász et al., 2024). In Malawi, for example, *S. mattheei* is a pervasive but underreported veterinary parasite of cattle and has been incriminated in both MGS and FGS (Stothard et al., 2025a). This places *S. mattheei* as the only parasitic helminth capable of inflicting disease within the human genital tract, uniquely arising from zoonotic transmission, that is, from infected snails via infected livestock. This brings a novel One Health dimension into the SRH agenda in Africa, where the close association between human health and agriculture is evidenced directly by this fascinating parasite. Raising its awareness within the public health sector is first required, before later intersectoral engagement with a variety of influential agricultural and environmental stakeholders (Stothard et al., 2025a).

## Kindling interest in genital tract parasites

As *Parasitology* commissions two special issues each year, with sponsorship in part from Cambridge University Press & Assessment, two related international meetings were organized by the authors, who hoped to kindle wider attention on parasites of the genital tract. Both meetings were held in September 2024; the first was with the British Society for Parasitology-Autumn Symposium (BSP-AS) in London, U.K., and then the second within the International Congress of Tropical Medicine and Malaria (ICTMM) in Kuching, Malaysia. A third scientific meeting was held independently in London in between the BSP-AS and ICTMM, led by LifeArc, with wider support from the Global Schistosomiasis Alliance (GSA) and partners from the Genital Bilharzia in Southern Africa (BILGENSA) Research Network. The LifeArc meeting was a hybrid scientific workshop with in-person and online attendees entitled ‘*Female Genital Schistosomiasis: Translational Challenges and Opportunities*’. Their ambition was to identify new partnerships and practical solutions which could better address the global challenge that surveillance and control of FGS pose.

Since this special issue was to take a more holistic approach to the study of parasites in the genital tract, inclusive of medical, veterinary and wildlife perspectives, submissions that addressed broader themes were also encouraged. In line with new communication initiatives for the journal (Ellis et al., 2025), to supplement several invited submissions, an open call was placed on the *Parasitology* website. Additionally, a short video was posted online (https://www.youtube.com/watch?v=NNGX2vurKVo) to describe the intention of the special issue in greater detail. This led to a total of 19 accepted manuscripts that formed a new literature with the following synopsis provided here.

## The use of gender and bovine vaccines

While sex and gender are very important determinants in the epidemiology of parasites of the genital tract, the statistics on how researchers use these terms within their studies have not been looked at in detail. In her review, Tadiri (2025) investigated the use of ‘gender’ within 174 studies published in *Parasitology* where the term was mentioned. Only 8·0% of papers correctly used the term, demonstrating persistent confusion between ‘sex’ and ‘gender’ terminology. Correct incorporation of the gender dimension, which should be considered for all human studies, requires greater effort, as it is a complex social variable. When a difference is found between men and women, it should be carefully considered whether that difference is likely to be due to biological (sex) or social (gender) factors (Tadiri, 2025). Put simply, parasitologists studying animals should simply refer to biological sex, which may not change from baseline to end-of-study assessments, whereas those studying humans should incorporate sex with more nuanced gendered terminology, cognizant of behavioural norms and healthcare access dynamics.

Despite encouraging submissions from veterinary and/or wildlife perspectives, only one was received. This addressed bovine trichomonosis, caused by *Tritrichomonas* (*Trit.*) *foetus*, a venereal disease of cattle, similar to human trichomoniasis. Santos et al. (2025) reminded us of its contemporary importance in cattle production in naturally mated animals, where bulls remain largely asymptomatic but mated cows become infected and then often infertile and/or abort their foetus. Alongside further adoption of artificial insemination methods, which diminish transmission, a whole-cell killed *Trit. foetus* vaccine formulation, using the Queensland isolate TfOz5 (vaccine strain), was explored to gain better control (Santos et al., 2025). Vaccinated animals were subsequently challenged by experimental infection with the TfOz-N36 (Northern Territory isolate). Whilst raised titres of anti-*Trit. foetus* IgG antibody was confirmed; its protective potency was modest. Thus, the search for an effective vaccination formulation in cattle continues, in parallel with vaccine research for human trichomoniasis (Smith and Garber, 2014).

## Focusing on male genital schistosomiasis

Surprisingly, there is no rapid diagnostic test or standard operating procedure to diagnose MGS, which was discussed by Neufeld et al. (2025). The diagnostic landscape for MGS faces a multitude of challenges and requires further efforts to improve performance and reduce knowledge gaps at all levels: sample collection, processing, storage and analysis. After deliberations, they proposed a two-step diagnostic algorithm, first with a serological blood test for antibodies against schistosome soluble egg antigens, and second by inspection of the ejaculate with either light microscopy or molecular DNA testing (Neufeld et al., 2025). Having two tests, the first with high sensitivity and the second with high specificity, could help in standardization of MGS diagnostics in future research and surveillance.

Clinical features of MGS were discussed by Richter et al. (2025), who used a combination of diagnostic tools, inclusive of ultrasound, as applied to four clinical cases who were children. In this paediatric setting, many clinicians are not fully alert to MGS, but sequelae include: hydrocele, hypogonadism, varicocele, cutaneous granulomata on the penis and scrotum, and echogenic spots in the prostate and the epididymis, alongside testicular masses. Some of this morbidity ameliorated after praziquantel treatment, though further clinical follow-ups were needed (Richter et al., 2025). The occurrence of MGS already in early childhood underscores the need to treat schistosomiasis at an early age to prevent unnecessary complications, which, if left untreated, may require surgery or may no longer become reversible.

A longitudinal study of MGS in men was reported by Mainga et al. (2025), who attempted to follow male participants over a calendar year in Southern Malawi. Diagnostics included light microscopy of urine and ejaculate, as well as the use of molecular species-specific assays. Wider testing for STIs, inclusive of *T. vaginalis,* took place (Mainga et al., 2025). Despite praziquantel treatment, MGS appears chronic, and while the majority were caused by *S. haematobium,* a sizeable minority included co-infections with *S. mattheei.* Effective transmission control of the latter remains a tangible challenge in future case management and control measures (Mainga et al., 2025). More broadly, the policies surrounding syndromic management of STIs in Malawi need to be reconsidered in how MGS often presents and is later triaged within primary care or SRH clinics.

Unlike FGS, there is a paucity of applied social science studies on MGS, such that many psychosocial and cultural dimensions are inadequately understood. Nyamwanza et al. (2025) conducted a scoping review of current literature in an original attempt to better contextualize MGS within men’s sexual and reproductive health and rights (SRHR). Men infrequently report to health services; hence, carefully addressing masculinity and associations with (sub)fertility is needed to overcome certain behavioural norms that are barriers to health seeking (Nyamwanza et al., 2025). They noted that everyone within a household could have schistosomiasis, from infancy onwards, and outlined a pragmatic social science research agenda for boys and men along the route to maturity.

## Genital schistosomiasis in migrants and travellers

Due to travel-related exposure, patients with schistosomiasis can report to clinics outside of endemic areas. In the U.K. and working from the Hospital for Tropical Diseases in London, Rafferty et al. (2025) discussed how the high levels of migration from, and travel to, sub-Saharan Africa are leading to a greater number of patients presenting with genital schistosomiasis. Given the overall lack of knowledge of genital schistosomiasis in non-endemic areas, understanding and consistency between individual clinicians of the investigations and management of patients with genital schistosomiasis is likely to be poor (Rafferty et al., 2025). In their article, they review current knowledge of genital schistosomiasis in non-endemic areas, available clinical management guidelines, and barriers to clinical care of patients. For example, easy access to praziquantel in the U.K. is bottlenecked, and the drug is surprisingly costly to procure. Of note, clinical management of FGS requires involvement of at least four different specialities: infectious diseases, sexual health, gynaecology and urology to fully investigate FGS, differential diagnoses and possible complications (Rafferty et al., 2025). This clearly poses a challenge in inpatient and outpatient coordination and oversight, being costly and slow.

Similarly, Roure et al. (2025) analysed the early results of a screening and management protocol of imported FGS in migrant women from endemic countries to Spain. Of note, all 136 women screened were from Senegal, and everyone had one or more clinically relevant genitourinary findings. This could include pelvic pain, vaginal discharge and menstrual disorders. Whilst praziquantel treatment was well tolerated and ameliorations were observed, future clinical trials are needed to optimize dosing and re-treatment schedules, alongside the implementation of better point-of-care diagnostics to explore the association between FGS and HPV and to prevent later risks of cervical cancer. The high prevalence of abnormal cytology with HPV-negative results observed among women was another indictment for closer scrutiny of *S. haematobium* and cervical dysplasia (Roure et al., 2025).

## Focusing on female genital schistosomiasis

A key challenge in schistosomiasis control is addressing its spatial focality, where adjacent villages, for example, can have widely different prevalence of infection, a phenomenon known as overdispersion, and detailed disease cartography is required. Tchuem Tchuenté et al. (2025) used a precision mapping approach in Cameroon to identify locations where UGS was present and therefore could better define local risk for FGS. Using disease surveillance in schools, the prevalence of egg-patent schistosomiasis was: 0% in Ayos, 8% in Akonolinga, 33% in Bertoua and 48% in Doume (Tchuem Tchuenté et al., 2025). From these precision mapping observations, the selection was made for the FGS study of Nyotue et al. (2025) to be based within the health districts of Bertoua and Doume.

Nyotue et al. (2025) assessed the acceptability and feasibility of FGS screening by comparing static HIV clinics versus mobile pop-up ones. To help with visual FGS diagnostics using a colposcope, a clinician was remotely trained to diagnose lesions, with success measured by expert comparison using cervix images. In this setting, 1242 women participated, and FGS lesions were noted in over half of the women examined (Nyotue et al., 2025). They determined that in mobile pop-up clinics, the acceptability of colposcopy was better, particularly for screening younger women. Furthermore, opportunities for remote diagnosis via telemedicine should be further explored where the expert staff pool within the local clinic is constrained.

Whilst medical services in primary care are relatively strong in Cameroon, Liberia is still recovering from civil war. This conflict catastrophically damaged both health infrastructure and staffing. Even so, schistosomiasis is well-known and endemic within inland regions, with attempts before COVID-19 to deliver regular praziquantel treatment to children in at-risk schools. As many women may unknowingly suffer from FGS, to help with initial treatment triage, a FGS scorecard was developed based on reported signs and symptoms. Bell-Gam Woto et al. (2025) appraised this FGS scorecard against a cursory point-of-care gynaecological examination undertaken by a midwife with a speculum, in conjunction with parasitological inspection of urine by microscopy (Bell-Gam Woto et al., 2025). From a total of 400 women examined, the overall prevalence of UGS and FGS was less than 10%, but the FGS scorecard was able to detect certain risk associations, for example, women who fished regularly were more likely to present with FGS, whereas those who lived > 15 km from a local river were less likely to present with FGS. In this resource-poor setting, using the FGS scorecard alone was judged sufficient to prioritize praziquantel treatment for those in most need.

A more detailed diagnostic assessment of FGS in Malawian women was undertaken by Kumwenda et al. (2025) over a 1-year clinical sub-study with three inspection time points, examining 86 women with a proven history of UGS. A detailed cervicovaginal examination with a portable colposcope was conducted, examining cervicovaginal lavage, cervical swabs, cervical biopsy and urine with traditional parasitological and molecular diagnostic methods. FGS appeared widespread locally, with infections with *T. vaginalis* and high-risk HPV noted (Kumwenda et al., 2025). Further to the detection of *S. haematobium*, a number of women had concurrent infection with *S. mattheei.* Despite an active national control programme in Malawi, improved prevention and management of FGS is needed. Dovetailing these services with local SRH clinics, alongside HIV/AIDS programmes, could be the basis of developing an appropriate holistic health intervention package within primary care (Kumwenda et al., 2025).

## Diagnostic improvement in female genital schistosomiasis

Clinical colposcopy is the ‘gold standard’ for detection and staging of FGS; however, the availability of sufficiently trained or accredited colposcopists is a bottleneck to progress. Changes in cervicovaginal surfaces associated with egg damage can be heterogeneous and subtle, such that classifications, even by experts, can be subjective, lacking specificity. Using an archival image bank, Lemin et al. (2025) explored the application of computer vision methods, based upon artificial intelligence algorithms, to better quantify and more formally standardize observed lesions. Several challenges were identified, particularly in relation to the image orientation of the cervix, that made accurate labelling problematic (Lemin et al., 2025). This may be overcome immediately with better annotation methods and model architectures. Whether these methods will eventually make it into automated machinery at the point-of-care, only time will tell, but the ability to more quickly screen and compare reference imagery has strong potential.

Despite the development of numerous rapid diagnostic tests for a variety of tropical diseases, light microscopy still has an important role to play in diagnostic services. Exploring this platform and developing a low-cost method in Malawi, Stothard et al. (2025b) demonstrated that schistosome eggs could be directly seen in sediments from cervicovaginal lavage. Moreover, upon incubation in freshwater, these eggs could be hatched to release miracidia that were then collected onto FTA^®^ cards for later genetic analysis by PCR to identify *S. haematobium, S. mattheei* and hybrids thereof (Stothard et al., 2025b). Of special note, one woman was seen to have more than 50 schistosome eggs within her cervicovaginal lavage, many of which were viable. This was evidence demonstrating that vaginal discharge or menstrual blood could represent a minor environmental transmission route, such that secure menstrual hygiene management is advised.

The search for better isothermal molecular methods to incriminate FGS and HPV at the point-of-care continues. Smith et al. (2025) reviewed current isothermal diagnostics, with a focus on recombinase polymerase amplification and recombinase-aided amplification, noting the ability to multiplex technologies. Reference molecular testing with real-time PCR assays is typically constrained in low-resource settings owing to the necessities of trained staff, suitable equipment, a reliable source of electricity, a stable cold-chain and sufficient funding (for staff and reagents). They suggested that isothermal molecular diagnostics will be more pragmatic for application in these basic settings in peripheral clinics (Smith et al., 2025). It would also seem that diagnostic testing of genital self-swabs would be the most suitable specimen source, with future possibilities for home-testing, before later referral to a local health facility with reference diagnostics or clinical colposcopy, if available.

## Harnessing critical advocacy and co-ordinated actions

The next four papers further highlight the research and control landscape for schistosomiasis, bringing FGS more to the fore. In so doing, each attempt to solidify the current rationale for existing international partnerships seeks to strengthen the ambition or resolve of those stakeholders yet to join. Using a broader health systems lens, the latter seeks to strike for a more equitable balance within the global SRHR agenda.

Oluwole et al. (2025) offered a perspective from the non-governmental development organization (NGDO) Sightsavers, which has a legacy of kick-starting public health actions on FGS in Liberia and Nigeria, for example, with the introduction and pilot use of the FGS Score Card. They take a broader vision on FGS, however, noting its interplay with UGS and MGS. Alongside improved case management for FGS, bolstering preventive chemotherapy by earlier and expanded access to praziquantel is the most sensible rationale to gain longer-lasting control by scaled-up prevention (Oluwole et al., 2025). However, this will not be easy to achieve or maintain with the recent drop in external USAID and DFID funding.

Mainstreaming FGS surveillance and its control into the routine health system is therefore a priority. This relies more on in-country advocacy and raising sufficient domestic or internal funding. Mutapi et al., (2025) share their expectations and experiences in Zimbabwe, placing its relevance with other African countries where UGS is endemic. To date, many countries simply lack programmatic guidance to achieve effective mainstreaming (Mutapi et al., 2025). Greater effort is therefore needed for intersectoral collaboration and coordination with better sharing of domestic resources, where a careful balance of interventions that address water, sanitation and hygiene improvements, environmental management, health education and inclusion of preschool-aged children is needed. It is here that the national schistosomiasis control programme must seek wider engagement and support across the health system, rather than standing alone with outside support. It needs to adapt towards more inclusive activities rather than just operating through vertical systems by offering praziquantel treatment, via spatially fluid community-based delivery initiatives, to provide adequate empowerment to those fixed-point peripheral health centres in primary care.

The NGDO with the greatest recent impact on the control of African schistosomiasis is Unlimit Health, formerly the Schistosomiasis Control Initiative (SCI), which was originally supported with a Bill & Melinda Gates Foundation start-up grant (Fenwick et al., 2009). Without the SCI and working with several (inter)national partners, it would be unlikely that control of schistosomiasis today would benefit from the Merck-KGa annual donation of 250 M tablets of praziquantel. This pharmaceutical gift is, of course, ring-fenced for treatment of school-aged children, as overseen by the WHO, and not to be utilized for other purposes where the Ministry of Health should take primary financial and procurement responsibilities.

Fleming et al. (2025) share their latest information on recent studies that attempt to better define the FGS burden, with more robust spatial estimates and age‐related risk data. They identified critical threats to future progress that they, and many others, would like to see. While praziquantel remains the cornerstone of preventive chemotherapy and individual treatment, single‐dose regimens appear insufficient, such that repeated dosing is needed (Fleming et al., 2025). The introduction of the paediatric formulation of praziquantel, where very young children can benefit from this medicine, is also an important facet of earlier control of FGS, though this medicine is not to be gifted by Merck-KGaA. The epidemiological link with mother and child is obvious, for if the mother was infected, her child will likely also be at raised environmental risk of FGS, as pointed out before (Bustinduy et al., 2017; Stothard, 2025). Integrating FGS care into SRH platforms, it is now essential to deploy at scale some of the developed training curricula, minimum service packages and community education and engagement tools. This could be another coordinating role for the WHO-Geneva or WHO-AFRO offices.

The final paper in this Special Issue arises from the LifeArc-led meeting entitled ‘*Female Genital Schistosomiasis: Translational Challenges and Opportunities*’. Wasson et al. (2025) harness the collective knowledge and wisdom of experts in the field who debated on research needs and priorities in FGS. Several practical solutions were identified, which formed a comprehensive research agenda that could drive appropriate actions on tackling FGS, and a series of discrete translational actions were developed (Wasson et al., 2025). A good example was the process to engage with the Ministries of Health and Education, and those providing care for women and girls with FGS in affected countries to agree on a strategic approach that creates standardized school and clinical curricula to improve FGS awareness among affected communities as well as within the medical profession for its prevention, diagnosis and treatment (Wasson et al., 2025). At the same time, consultation and collaboration with local communities in endemic areas are needed to create appropriate public health messaging on FGS, disseminating it through multiple platforms that improve awareness across multiple ages and groups. To achieve this, it is evident that the journey ahead is costly and decades-long. No doubt there will be several intrinsic and extrinsic hurdles in the way, but the signposting towards this journey’s end is now much clearer.

## What future for parasites of the genital tract?

It is apparent that our Special Issue features schistosomiasis most strongly, with briefer considerations on general parasitological research on ‘gender’, co-infections with trichomoniasis and vaccine development for the cattle parasite *Trit. foetus*. Whilst this body of evidence sets a new yardstick, we remain mindful of those medical parasites absent from our list (e.g. *Entamoeba histolytica, Giardia duodenalis, Toxoplasma gondii, Trypanosoma cruzi* and not forgetting several ectoparasites) that are linked in some way with the genital tract, sexual transmission and/or congenital infections. We therefore encourage further attention on them in the future. By the same token, it is obvious that there are very few researchers studying similar parasites in the veterinary and wildlife sectors, perhaps due to a dearth of laboratory models, appropriate funding and academic positions to do so. Fascinating parasites of birds, oviduct flukes within the genus *Prosthogonimus* or the poultry protist *Trichomonas gallinae* or even brood parasitic cuckoos, were overlooked. Similar parasites in invertebrates, for example, oxyurid nematodes in cockroaches and beetles or microsporidia in crustaceans, were totally eclipsed. It would be too harsh for our readers to judge these omissions as purposeful, for they simply await another editorial impetus to feature them in a later special issue or commissioned review. To close, we hope you find ‘*Parasites of the genital tract: short- and long-term consequences*’ an informative introduction to this aspect of parasitic lifestyles and contemporary research on parasites.

## Supporting information

Stothard et al. supplementary material Stothard et al. supplementary material

## Supplementary material

The supplementary material for this article, the video clip mentioned in Figure 2, can be found at https://doi.org/10.1017/S0031182025101303.
